# Seabird-derived nutrients influence feeding pathways and body size in cryptobenthic reef fishes

**DOI:** 10.1098/rspb.2025.0539

**Published:** 2025-07-09

**Authors:** Laura-Li Jeannot, Juan Pablo Lozano-Peña, Anna Zora, Simon J. Brandl, Nicholas A. J. Graham

**Affiliations:** ^1^ Lancaster Environment Centre, Lancaster University, Lancaster, UK; ^2^ Department of Marine Science, Marine Science Institute, The University of Texas at Austin, Port Aransas, TX, USA; ^3^ Fregate Island Foundation, Roche Caiman, Seychelles

**Keywords:** seabird guano, nutrient subsidies, coral reef ecology, nutrient enrichment, stable isotope analysis

## Abstract

Cross-ecosystem nutrient transfer can enhance coral reef functioning in an otherwise oligotrophic environment. While the influence of seabird-derived nutrients on coral reef organisms is increasingly recognized, how they are integrated into reef food webs remains unclear. Cryptobenthic reef fishes are crucial for energy transfer on coral reefs, and their fast life histories imply that they respond strongly to seabird-derived nutrients. Here, we investigate how variation in nearshore seabird nutrient subsidies affects coral reef fish communities. By comparing fish communities across locations differing in seabird nutrient inputs and using stable isotope analysis, we explore nutrient integration across depth, their influence on cryptobenthic and associated larger reef fishes and investigated the relative reliance of cryptobenthic fishes on seabird-enriched benthic and non-enriched pelagic pathways. We find that, near seabird colonies, cryptobenthic fishes’ diets can transition from pelagic to benthic dominance; cryptobenthic fish communities are larger; herbivores and all feeding groups comprising potential cryptobenthic fish predators have higher biomass. Collectively, our results stress the importance of seabirds in shaping energy pathways and suggest that, even in dynamic, ocean-swept reef systems, cryptobenthic fishes can mobilize seabird subsidies and potentially act as a nutritional bridge to higher trophic levels.

## 1. Introduction

Nutrient transfer across ecosystems can significantly affect recipient communities by enhancing biomass [1], diversity [2] and productivity [3]. This, in turn, may influence system-wide trophic interactions and ecosystem dynamics [4,5]. Mobile consumers play a vital role in cross-ecosystem connectivity by actively translocating nutrients across ecosystem boundaries, and they are especially important when subsidizing otherwise nutrient-poor areas with allochthonous inputs from highly productive areas [6,7]. Although these nutrient flows are globally important drivers of both biomass and biodiversity, anthropogenic disturbances have severely disrupted many cross-ecosystem nutrient subsidies. Specifically, human-driven declines in mobile predators, changes in their distribution and feeding ecology through climate change and habitat degradation have dramatically altered nutrient cycling worldwide [8,9]. Understanding how ecosystems respond to such changes in nutrient vectors and the landscapes they traverse is of critical importance to efforts that aim to maintain and enhance local functioning and productivity.

Straddling the interface between land and sea, seabirds play a unique role as mobile consumers that transfer nutrients from their pelagic foraging grounds to the islands where they roost, via the excretion of nitrogen- and phosphorus-rich guano [10–12]. Therefore, they can reduce the frequently stringent nutrient limitations in these food webs and lead to increased biomass, stability and resilience [13,14]. However, their persistence is threatened by a range of anthropogenic pressures, including the frequent colonization of seabird islands by invasive predatory rats, which have caused global declines in seabird populations [15].

Coral reefs exhibit enhanced ecosystem functioning and productivity in response to seabird-mediated nutrient enrichment. Recent studies have demonstrated higher growth rates of algae, corals and reef fishes, as well as faster coral recovery after disturbances, and increased rates of key ecosystem functions [1,13,16–19]. To date, most knowledge of seabird-subsidized reefs is from low-lying atoll islands, with extensive, enclosed shallow-water reef habitats. Conversely, how nutrients from seabirds are incorporated into fringing coral reefs from high islands that drop rapidly into deeper water is poorly understood. While seabird subsidies can be integrated by organisms near islands with steeper reef slopes [12], nutrients also display variable residence times in rapidly flushing fringing reefs, raising questions about their integration by reef organisms and contrasts with other reef types [20,21]. In such systems, local point-source nutrient inputs can be significant [22–25] and are also complemented by deep-water nutrient resources and offshore pelagic subsidies [26–28]. Advancing our understanding of nutrient dynamics across diverse habitats is especially important as nutrient imbalance can have far-reaching implications for coral reef functioning [29].

Coral reef productivity is of vital importance for the societies that depend on them, with millions of people relying on coral reef fisheries for their livelihoods and food security [30]. The drivers of coral reef productivity are multifaceted and context-dependent [31,32], but small-bodied reef fishes with high turnover rates have recently been highlighted as a potentially critical consumer group in transferring energy and nutrients to higher trophic levels [33]. Characterized by their small size (generally less than 5 cm), these ‘cryptobenthic’ fishes often feed on poorly accessible food sources and represent an important source of readily available and steadily renewed nutrients for larger consumers, contributing up to 60% of the consumed biomass on coral reefs [33–35]. In spite of this critical importance for coral reef productivity, studies focusing on cryptobenthic fish have long been hampered by their small size and cryptic nature, and knowledge about cryptobenthic fish ecology remains fragmented [36]. Importantly, their extreme mortality rates and unique demographic dynamics render them highly responsive to local environmental changes [33,37]: differences in community composition, diet, individual weight, growth and biomass production have all been observed in relation to environmental variation, making them suitable indicator organisms for subtle environmental gradients [38]. This may include a strong response to localized changes in nutrient availability, seabird-derived or otherwise, with potential consequences on the relative importance of energy pathways used by cryptobenthic fishes.

While smaller fishes are typically associated with higher reliance on benthic pathways, there is evidence that background nutrient concentrations, benthic and pelagic productivity, distance to nutrient sources and depth can all influence the relative contribution of benthic and pelagic resources to marine organisms’ diets [39–41]. For example, over-enriched eutrophic systems can lead to benthic fish species shifting further to pelagic resources [39], but very little is known about how consumers respond to enrichment in nutrient-poor waters such as coral reefs. Previous studies suggest that seabird subsidies positively affect the quality of benthic resources such as turf algae, resulting in dietary behaviour changes in territorial damselfish [42], and can also lead to short-term increases in algal cover [16]. In parallel, seabirds can also stimulate plankton productivity, thereby fertilizing pelagic food webs [11]. However, it is currently unknown whether seabird-vectored nutrients can influence the relative importance of these energy pathways within marine organisms’ diets. Many fish species, including cryptobenthic fishes, are capable of utiizing both pelagic and benthic resources [36], and prey selection on the basis of resource quality and availability is widely established in reef fishes [43]. This raises the question of whether cryptobenthic fishes may shift resource use towards higher reliance on either of the seabird-subsidized pathways near seabird colonies. Cryptobenthic fishes’ highly restricted home ranges (<5 m^2^ [44]), and rapid life cycles are especially useful in the context of fringing reefs as they (in contrast to functional groups examined elsewhere [1]) may mobilize nutrients that would otherwise be quickly flushed and may reveal whether relative reliance on trophic pathways owing to seabird inputs is influenced by depth in these systems [39].

Here, we examine how variability in seabird-derived nutrient subsidies may affect cryptobenthic fish communities across a depth gradient. Specifically, using a natural gradient of seabird-derived nutrient availability across two sites at Fregate Island, Seychelles, coupled with stable isotope and community analyses, we investigate whether:

(i) seabird-derived nutrients are incorporated by cryptobenthic fishes near seabird colonies;
(ii) the relative importance of energy pathways used by cryptobenthic fishes is influenced by seabird nutrient enrichment;
(iii) cryptobenthic fishes exhibit community-level (community composition, density, species richness and biomass) and species-level (total length and weight) responses to differences in their nutrient environment; and
(iv) larger fish biomass is greater where seabird nutrient signals are higher.

## 2. Methods

### (a) Study site

**Figure 1.**
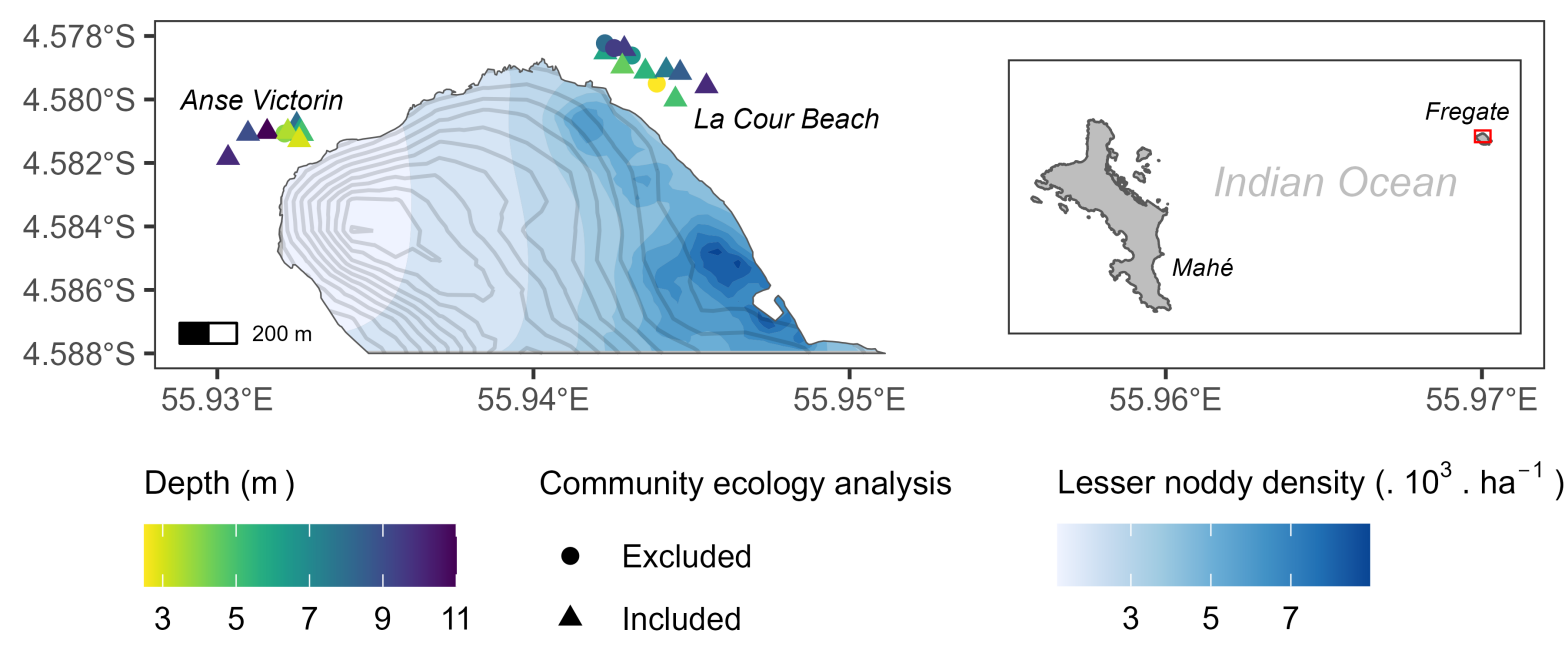
Map of the sampling locations. Lesser noddy density, the most abundant bird species in Fregate Island by an order of magnitude, was two-dimensional interpolated using the R package gstat v 2.1-2 [48] based on the 2022 census (A. Zora 2022, personal communication). Grey contour lines represent 10 m interval elevation changes.

Sampling was conducted on coral reefs around Fregate Island, located in the inner islands of the Republic of Seychelles (4°35' S, 55°56' E). Wildlife conservation initiatives commenced on the island in the 1970s: feral cats (*Felis catus*) were eradicated from Fregate Island in 1982, as they adversely impacted bird populations [45]; Norway rats (*Rattus norvegicus*), first documented in 1995, proliferated until an eradication programme was implemented in 2000 [46]. Post-eradication populations of seabirds increased from an estimated 2700 pairs of lesser noddies (*Anous tenuirostris*) in 1997 and 3030 white tern nests (*Gygis alba*) in 1999 to 229 304 and 11 325, respectively in 2022 [47]. Lesser noddies reside on the eastern side of the island, with no historically documented nesting activity on the western side, while white terns are found across the island (A. Zora 2022, personal communication; [47]; figure 1; electronic supplementary material, figure S1). Two locations were selected to represent varying levels of seabird-derived nutrient inputs: high nutrient input from seabirds occurs off the eastern beach at La Cour (hereafter seabird-rich location), given the high concentration of seabirds, a gentle seaward slope favouring nutrient run-off to the reef during rainfall, and the presence of groundwater deposits, while low seabird-derived nutrient input occurs at Anse Victorin (hereafter seabird-poor location), a beach at the bottom of a steep hill in an area largely devoid of seabirds (figure 1; rainwater run-off pictured in the electronic supplementary material, figure S2). The sampling locations feature a southwesterly current at La Cour and a westerly current at Anse Victorin.

### (b) Field sampling

For each sampling station, a coral outcrop was selected based on estimated size (150–350 cm curved surface length (CSL)) and sampling suitability for cryptobenthic fish sampling (e.g. preference for somewhat isolated, dome-shaped structures). Three photographs were taken using a randomly placed 0.5 × 0.5 m quadrat, and CSL was measured with a 10 m tape measure. A mesh net (hole size: approx. 1 mm) and an 8.5 m^2^ circular tarpaulin, both weighted with 5 kg metal chains wrapped around their base, were then laid over the outcrop. The coral bommie under the tarpaulin was then sprayed with a solution of 1 : 5 clove oil (Jedwards International, USA) to 95% ethanol. After approximately 5 min or when fish started falling at the bottom of the outcrop, the tarpaulin was removed. Four divers collected fish underneath the net, placing them in clear slider storage bags and gradually peeling the net back until the net was entirely removed and no fish were found for another approximately 5 min. All fish were then brought back to the surface and preserved in an ice bath until processing. For each site, three samples of turf algae and filter-feeding sponge (*Hyrtios erectus*) were collected within a 25 m perimeter of the coral outcrop as proxies for benthic (turf algae) and pelagic (sponge) sources of energy and nutrients [49,50]. In total, cryptobenthic fishes were sampled across 21 sampling stations (seabird-rich location: 12 stations, seabird-poor location: nine stations) ranging from 2.5 to 11 m depth following established protocols [51,52]. Furthermore, a 15 m transect was conducted at each station to record visually conspicuous fish species and habitat, respectively (total *n* = 19). Visually conspicuous fish were recorded using underwater visual censuses (UVC), with one diver recording species and size along a 15 m transect extending 2.5 m on either side (75 m^2^). In the laboratory, a high-quality photograph was taken of each cryptobenthic fish individual, before measuring and weighing each fish using digital callipers and a 0.001 g precision balance. Three species of cryptobenthic fishes were selected for stable isotope analysis based on local abundance, frequency of occurrence and expected dietary differences: *Enneapterygius abeli* (Tripterygiidae)*, Eviota guttata* (Gobiidae) and *Cirripectes filamentosus* (Blenniidae; electronic supplementary material, table S1). Up to three specimens of each species were taken per station and oven-dried at 60°C for 24 h. All other samples were preserved in 95% ethanol. To account for habitat complexity, an index was estimated using an 8 × 8 grid superimposed on the three pictures taken during sampling. Each square was attributed a score of 1 if it contained high-complexity microhabitats (overhang, branching coral, hole) or 0 if not, totalling a collective score for each photo that ranged between 0 and 64. This value was then averaged across the three pictures to yield a complexity index for each station.

### (c) Stable isotope analyses

Both sources (turf algae, sponge) and the three fish species were used for dual bulk carbon *δ*^13^C and nitrogen *δ*^15^N stable isotope analysis. Samples were used to establish differences in nutrient input across the two locations, with the assumption that higher nitrogen values correspond to seabird-derived nutrient uptake reflecting the high tropic levels at which seabirds feed [1].

To avoid potential bias owing to gut content in the isotopic values of the consumers, the anterior portion of the fish was removed after drying. Samples were ground to a fine powder using mortar and pestle. For the fish and source samples, approximately 0.5 and 1.35 mg, respectively, were weighted and sealed into tin capsules. Thereafter, carbon and nitrogen isotope compositions of source and fish samples were measured at The University of Texas at Austin (Marine Science Institute, Core Isotope Facility in Port Aransas, TX, USA), via combustion in an automated system for coupled nitrogen- and carbon-isotope measurements using a Thermo Fisher Scientific Flash EA-Isolink CNSOH elemental analyser connected to a Thermo Fisher Scientific Delta V Plus isotope-ratio-mass-spectrometer. Analytical precision was calculated as the mean within-run s.d. from United States Geological Survey (USGS)-40 and USGS-41a and was less than 0.2‰ for both carbon and nitrogen isotope composition. Stable isotope values are reported using standard delta (*δ*) notation in parts per thousand (‰), as described in the following equation:


$$\delta X=\left(\frac{R_{sample}}{R_{standard}}-1\right)\times 1000,$$

where X is ^13^C or ^15^N and *R* is the ratio of the heavy to light isotope (i.e. ^13^C/^12^C or ^15^N/^14^N) of the sample and international standards (Vienna Pee Dee Belemnite (V-PDB) and atmospheric nitrogen (AIR) for carbon and nitrogen, respectively; [53,54]). Consumer C : N ratios indicated samples had less than 5% of lipid content (mean C : N ratio = 3.20 ± 0.05 s.d.), thus eliminating any potential bias owing to high lipid content [55]. Acid fumigation had negligible effects on *δ*^13^C, and we therefore used non-acidified samples in our analyses (see the electronic supplementary material, table S2, for details).

### (d) Data analysis

First, we compared the *δ*^15^N values in source materials to confirm that the two chosen sampling locations differed in their seabird-derived nutrient input. To do so, a Bayesian linear mixed-effects model was first used with separate levels for each source and their location as fixed effects, using site as a random effect and with *δ*^15^N as the response variable. We then investigated the effect of depth on source isotopic signals, running two separate models for each source to avoid overparameterization. As we expected the effects of depth to depend directly on proximity to seabirds (i.e. guano dissipation in the seabird-rich location), we used the interaction between depth and location as a fixed effect and site as a random effect. All models were run with a Gaussian error distribution, using four chains with 10 000 iterations including 5000 burn-in samples. Model validation was performed using visual evaluation of chain convergence and Geweke and Gelman diagnostics (electronic supplementary material, table S3). We used posterior parameters to predict values across a sequence of 100 evenly spaced values within the sampled depth range (2.5–11 m). This was repeated for 1000 draws, and each predicted model fit was plotted alongside raw source isotope data. The same process was repeated for *δ*^13^C values.

All analyses involving cryptobenthic as well as larger fish species are presented in the electronic supplementary material, table S4, along with the hypothesis being tested. All Bayesian models were run with the same model specifications as for the sources as well as default priors unless specified in the electronic supplementary material, table S4, in which case prior predictive checks were performed. For hypotheses 1 and 2, no depth model was fitted for *C. filamentosus*, given that there was no overlap in depth between samples from the seabird-rich and the seabird-poor locations. Similar to sources, posterior parameters were used to predict values across a sequence of 100 evenly spaced values within the sampled depth range for each cryptobenthic fish species for 1000 draws. Given that size can influence *δ*^15^N in fishes and that ontogenetic diet changes could imply variation in *δ*^13^C, additional models were run taking into account fish total length as a fixed effect. To investigate whether seabird nutrients can influence benthic or pelagic pathway use in cryptobenthic fish, we estimated the reliance of cryptobenthic fishes on benthic and pelagic sources, using single-biotracer (*δ*^13^C) isotope mixing models with the R package MixSIAR [56]. Turf algae and sponge were used as sources to represent reliance on benthic and pelagic pathways, respectively. We chose a trophic discrimination factor (TDF) of 0.4 ± 1.3‰ s.d. for *δ*^13^C, which is considered widely applicable in aquatic food webs [57] and which was selected instead of more recently proposed TDFs after a sensitivity analysis ([58]; electronic supplementary material, table S5). Depth was used as a continuous variable, under the assumption that reliance on nutrients of pelagic origin increases with depth [59]. Model parameters were set using the pre-defined ‘extreme’ parameter set, corresponding to three chains of 3 000 000 iterations with 1 500 000 burn-in samples and a thinning interval of 500. Diagnostics are reported in the electronic supplementary material, table S3. As non-diet directly related factors, including shifts in the isotopic compositions at the base of food webs [60], may obscure the interpretation of foraging habits derived from the bulk isotopic composition of organismal tissues, we used location-specific C isotope ratios of sources in our Bayesian mixing models to account for inter-location variability in isotopic baselines. Sponges can exhibit spatiotemporal variations in diets, and consumption of benthic material cannot be ruled out, suggesting they do not reflect an absolute pelagic signal but rather a more pelagic signal relative to turf algae [50]. Therefore, only relative diet estimates of cryptobenthic fishes are reported.

For the community analyses, five stations out of 21 were excluded owing to high-swell conditions and resulting concerns about the comprehensiveness of the collection. An additional station was excluded owing to the presence of large schools of *Parapriacanthus guentheri* (Pempheridae) and *Verulux cypserulus* (Apogonidae), which were not found anywhere else. Therefore, while individuals from the 21 stations (full set) were used for stable isotope analyses as swell is assumed to have no impact on isotopic composition, only 16 stations were kept for community analyses (reduced set). Comparability between the two locations was established by contrasting both habitats and microhabitats. A PERMANOVA (999 permutations) was performed to identify differences in habitats found in the transects, and microhabitat complexity indices as defined above were averaged and compared across the seabird-rich and the seabird-poor locations.

To identify changes in community composition among the 16 remaining stations (seabird-rich location: 8; seabird-poor location: 8), we first performed a redundancy analysis (RDA) using location and depth as constraints. As changes in fish size have been reported following nutrient enrichment by seabirds [19], total length and weight were investigated for (i) all individuals, with species as a random effect, and (ii) the three overall most abundant species (>20 individuals at each location): *Eviota bipunctata, Ev. guttata* and *En. abeli*. Differences between pairwise effect levels were explored using post hoc contrasts. To investigate potential differences in larger reef fish communities, UVC data were used to compare visually conspicuous fish biomass across the two locations. For each site, biomass was calculated using length–weight relationships from FishBase Bayesian estimators [61,62]. To investigate the potential cascading effects of seabird nutrients being transferred to larger consumers, we partitioned visually conspicuous fishes into seven feeding groups based on the literature: herbivores, omnivores, mixed carnivores, invertivores, piscivores, corallivores and planktivores. We then computed biomass for each group. Herbivores were compared to all other groups to explore whether depth had diverging effects on biomass according to feeding group.

## 3. Results

The two locations were comparable in terms of their benthic habitat and complexity. Coral cover was similar (seabird-rich location: 55 ± 9% s.d.; seabird-poor location: 54 ± 18% s.d.), with the PERMANOVA revealing no difference in benthic composition between the two locations (location: *F*_1_ = 1.953, *R*^2^ = 0.932, *p* = 0.107). Station complexity ranged from 6 to 63, but average complexity of the sampled stations was comparable, with 33.7 ± 16.2 s.d. and 40.4 ± 15.3 s.d. for Anse Victorin and La Cour, respectively.

### (a) *δ*^15^N: seabird nutrient enrichment

**Figure 2.**
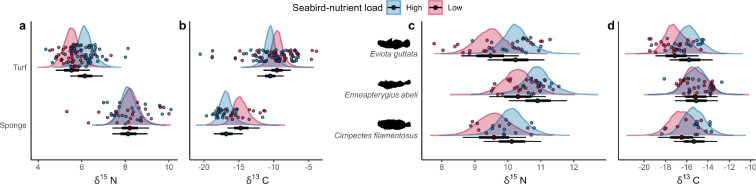
(a) *δ*^15^N and (b) *δ*^13^C of turf algae and sponge. (c) *δ*^15^N and (d) *δ*^13^C of three cryptobenthic fish species. Density curves and caterpillar plots (50 and 95% CIs) represent fitted values from Bayesian linear models based on 1000 posterior draws. Dots represent raw values.

We found consistently higher *δ*^15^N values in the benthic end-member (turf) in the seabird-rich location, suggesting that nutrients originating from seabird guano are transferred to nearshore habitats on the eastern side of the island, and either not or relatively less to the seabird-deprived western side (turf algae *δ*^15^N: 6.13 ± 0.68‰ s.d. in the seabird-rich location and 5.54 ± 0.55‰ s.d. in the seabird-poor location). The model confirmed this trend, with a posterior probability of higher *δ*^15^N in the seabird-rich location greater than 99% (figure 2a; electronic supplementary material, table S7). There was moderate evidence of a divergent effect of depth on N isotopic composition of turf in the two locations, with a slope estimate of −0.06 and 95% credible intervals (CIs) (−0.13; 0.02) in the seabird-rich location and a slope estimate of 0.02, with 95% CI (−0.05; 0.09) in the seabird-poor location, suggesting *δ*^15^N may decrease more with depth near seabirds (electronic supplementary material, figure S3). More specifically, the largest difference in *δ*^15^N across the two locations occurred at the shallowest sampled depths (approx. 3 m), but was broadly comparable at approximately 10 m, allowing us to partition these locations into high seabird-nutrient load (corresponding to the seabird-rich location La Cour) and low seabird-nutrient load (corresponding to the seabird-poor location Anse Victorin) locations. By contrast, the lack of ^15^N enrichment of the pelagic-feeding source in the seabird-rich location (sponge *δ*^15^N: 8.20 ± 0.88‰ s.d. and 8.21 ± 1.21‰ s.d. in the seabird-rich and seabird-poor locations, respectively) demonstrates that any further seabird-derived nutrient uptake would be of benthic origin, therefore enabling us to differentiate benthic and pelagic energy pathways to indicate integration of seabird nutrient subsidies into food webs.

All three cryptobenthic fish species exhibited a consistent pattern of elevated *δ*^15^N in the seabird-rich location (figure 2c; table 1). *δ*^15^N values ranged from 7.77 (*Ev. guttata*) to 12.28‰ (*En. abeli*). Average *δ*^15^N reflected the species’ feeding groups: *δ*^15^N was lowest for the mixed herbivorous *C. filamentosus* (9.83 ± 0.62‰ s.d.)*,* followed closely by the presumably mixed-feeder *Ev. guttata* (which also showed the highest variability (9.91 ± 0.908‰ s.d.))*,* while the invertivorous *En. abeli* presented the highest *δ*^15^N value (10.6 ± 0.526‰ s.d.). Regardless of baseline *δ*^15^N differences, the model provided support for higher *δ*^15^N near seabirds for all three species, with posterior probabilities of 99, 98 and 93% for *Ev. guttata, En. abeli* and *C. filamentosus*, respectively. When accounting for total length, the posterior probabilities of higher *δ*^15^N near seabirds remained 99 and 98% for *Ev. guttata* and *En. abeli* and were slightly lower for *C. filamentosus* at 79%. Individual species models integrating depth for *Ev. guttata* and *En. abeli* failed to reveal a consistent trend of depth dependence for *δ*^15^N (electronic supplementary material, figure S4).

**Table 1.** High (seabird-rich)—low (seabird-poor) locations contrast table for *δ*^15^N, *δ*^13^C, length and weight. (The estimate column represents the mean estimated difference between high and low seabird-nutrient locations for each variable. Lower HPD and upper HPD provide the 95% highest posterior density intervals and represent the range within which the true effect is most likely to fall with 95% probability. The post.prob column represents the posterior probability (from Bayesian hypothesis testing) that the effect for ‘high’ is greater than the effect for ‘low’ for each variable. Higher values indicate stronger evidence in favour of ‘high > low’.)

variable	species	estimate	lower HPD	upper HPD	post.prob
*δ*^15^N	*Ev. guttata*	0.78	0.17	1.37	0.99
	*En. abeli*	0.61	0.00	1.2	0.98
	*C. filamentosus*	0.54	−0.23	1.28	0.93
*δ*^13^C	*Ev. guttata*	1.37	0.04	2.77	0.98
	*En. abeli*	0.13	−1.27	1.47	0.56
	*C. filamentosus*	1.16	−0.4	2.93	0.93
length	*Ev. bipunctata*	0.15	0.01	0.3	0.98
	*Ev. guttata*	0.17	0.01	0.34	0.98
	*En. abeli*	0.05	−0.08	0.18	0.80
	all species	0.1	0.00	0.2	0.97
weight	*Ev. bipunctata*	0.43	−0.02	0.89	0.97
	*Ev. guttata*	0.47	−0.02	1.00	0.96
	*En. abeli*	0.12	−0.27	0.57	0.73
	all species	0.24	−0.04	0.53	0.96

### (b) *δ*^13^C: benthic and pelagic-feeding pathways

*δ*^13^C values suggested two distinct carbon pathways: sponges, as filter feeders, exhibited a more pelagic carbon signature (−16.0 ± 2.38‰ s.d.), while turf algae had a signature considered to be more representative of benthic-derived carbon (−10.2 ± 3.15‰ s.d., respectively). In contrast to *δ*^15^N values, sponges displayed a more marked difference between the two locations, with the seabird-rich location associated with higher *δ*^13^C values or a more pelagically derived isotope signature (figure 2b; electronic supplementary material, figures S5 and table S7). Cryptobenthic fishes’ C isotope composition for all three species tracked closer to the sponge signal rather than the algae signal, even in the case of the algal-feeding *C. filamentosus* (−15.5 ± 1.45‰ s.d.) (*Ev. guttata* (−16.9 ± 1.49‰ s.d.); *En. abeli* (−15.0 ± 1.31‰ s.d.)). Both *Ev. guttata* and *C. filamentosus* exhibited an increase towards a benthic signature in the seabird-rich location (figure 2d; table 1), and this remained the case for *Ev. guttata* when accounting for total length (97% posterior probability). *δ*^13^C values for *En. abeli* decreased with depth, and this shift was more pronounced in the seabird-rich location (slope of −0.91 with 95% CI (−0.80; −0.17); electronic supplementary material, figure S6).

**Figure 3.**
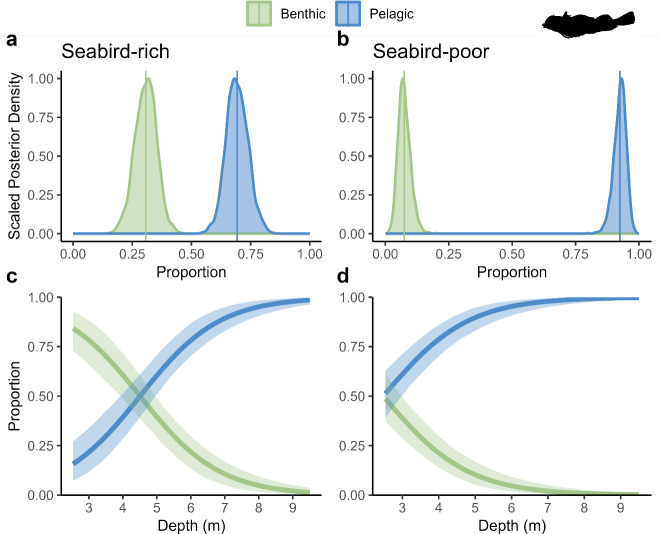
Posterior distributions for diet proportions for *En. abeli*. (a) and (c) represent scaled posterior density for overall diet proportions and diet proportions according to depth, respectively, in the seabird-rich location. (b) and (d) represent scaled posterior density for overall diet proportions and diet proportions according to depth, respectively, in seabird-poor location. Lines represent posterior medians, and shading represents the 90% CIs. Posterior distributions for diet proportions at different depth strata are available in the electronic supplementary material, figure S8.

The isotope mixing models shed additional light on pathway use across species and locations: *En. abeli* and *Ev. guttata* used 23 and 5% more seabird-enriched benthic food resources on average in the seabird-rich location, respectively (figure 3a,b; electronic supplementary material, figure S7). At the shallowest depths (3 m), where differences in seabird nutrient input between the two locations are highest, *En. abeli*’s diet was pelagically dominated in the seabird-poor site but benthically dominated in the seabird-rich site, with up to 36% more benthic resources consumed near seabird colonies, with a similar pattern emerging for *Ev. guttata*, with a 14% relative increase in benthic resource use near seabirds (electronic supplementary material, figure S7).

### (c) Community-level responses

Six cryptobenthic fish families were found across the two locations (Gobiidae, Tripterygiidae, Blenniidae, Apogonidae, Plesiopidae and Gobiesocidae), with a total of 770 cryptobenthic fishes from 72 species among all 21 stations (entire set) and 558 fishes across 39 species from the 16 stations that were retained for community analyses (reduced set). Overall, communities from both locations were similar (table 2). Predicted values for the species density and individual density models were slightly lower in the seabird-rich location; however, biomass predicted values were almost identical across the two locations (table 2; electronic supplementary material, figure S9). The RDA revealed no community-level compositional dissimilarity between seabird-rich and seabird-poor locations but an effect of depth (*p*-value_Location_ = 0.741, *p*-value_Depth_ = 0.007, *R*^2^_adj_ = 0.147; electronic supplementary material, figure S10). This was driven by *Ev. bipunctata* and *En. abeli*, which accounted for 48 and 43%, respectively, of variation along the first RDA axis and which were more abundant at shallow depths.

**Table 2.** Cryptobenthic fish community observed and predicted community indices. (Uncertainty refers to s.d., and numbers in brackets refer to 95% CIs for observed and predicted values, respectively.)

seabird nutrient input	density	species richness	biomass (g m^−2^)	Shannon diversity	weight (g)	total length (cm)
low (observed)	10.8 ± 6.0	3.3 ± 2.0	1.8 ± 1.3	2.0 ± 0.2	0.2 ± 0.1	17.9 ± 3.0
low (predicted)	12.3 (2.7; 23.5)	3.6 (0.9; 7.0)	2.6 (0.2; 6.2)			
high (observed)	9.2 ± 5.8	2.9 ± 1.6	2.4 ± 2.8	1.9 ± 0.3	0.2 ± 0.2	19.8 ± 3.1
high (predicted)	10.4 (2.7; 19.0)	3.4 (1.1; 6.4)	3.0 (0.4; 6.9)			
total (observed)	10.0 ± 5.8	3.1 ± 1.8	2.1 ± 2.1	1.9 ± 0.2	0.2 ± 0.1	18.9 ± 3.1

### (d) Total length and weight

**Figure 4.**
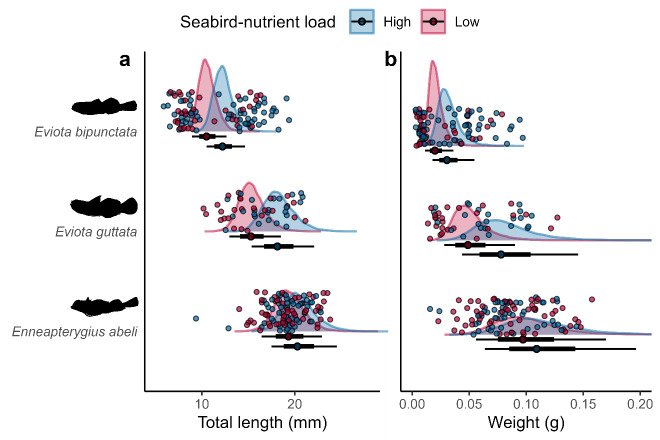
(a) Total length and (b) weight of three cryptobenthic fish species. Density curves and caterpillar plots (50 and 95% CIs) represent fitted values from Bayesian linear models. Dots represent raw values.

The community-level total length and weight models provided strong evidence for larger fishes in the seabird-rich location (97 and 96% posterior probability for higher total length and weight, respectively, in the seabird-rich location; table 2). Given that not all species were common enough for robust species-level comparison, we also modelled the three most abundant species at both locations: *En. abeli* (*n* = 111), *Ev. bipunctata* (*n* = 100) and *Ev. guttata* (*n* = 54). For both *Eviota* species, total length and weight appeared higher in the seabird-rich location (table 1; figure 4). These effects were less clear for *En. abeli*.

### (e) Associated larger reef fish biomass

**Figure 5.**
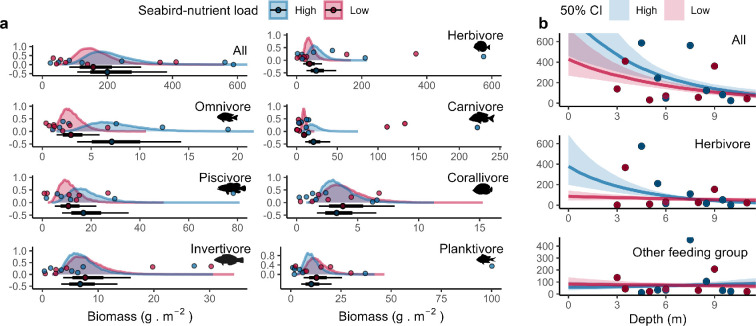
(a) Visually conspicuous fish biomass according to feeding groups and seabird nutrient load. Density curves and caterpillar plots (50 and 95% CIs) represent fitted values from Bayesian linear models. Dots represent raw values. Fish silhouettes depict the species with the highest biomass present in each feeding group. (b) Relationships between biomass and depth according to feeding groups and seabird nutrient load. Shaded bands represent 50% CIs.

In terms of mobile, conspicuous reef fishes, species composition was broadly comparable between the two locations and across depth (*p*-value_Location_ = 0.623, *p*-value_Depth_ = 0.196, *R*^2^_adj_ = 0.035; electronic supplementary material, figure S11), but biomass appeared slightly higher in the high seabird nutrient site, though with high uncertainty (posterior probability of 71%; electronic supplementary material, table S8; figure 5a). Partitioning of fish into feeding groups revealed that herbivores, mixed carnivores, omnivores and piscivores followed a similar pattern, with posterior probabilities that fish biomass was higher in the seabird-rich location ranging from 80 (herbivores) to 98% (carnivores). On the contrary, there was little effect of seabird nutrient inputs for invertivores, planktivores and corallivores, with posterior probabilities comprised within 32 (planktivores) and 40% (invertivores; electronic supplementary material, table S8). Our results suggested that biomass decreased with depth (slope estimate of −0.20, with 95% CI (−0.46; 0.04) and posterior probability of negative slope of 95% in the seabird-rich location; slope estimate of −0.15, 95% CI (−0.37; 0.06) and posterior probability of 93% in the seabird-poor location; figure 5b). This was mainly driven by an increase in nearshore herbivore biomass, while non-herbivorous biomass remained largely unchanged with depth. For herbivores specifically, seabirds had a divergent effect on how depth influenced fish biomass, with shallow-water biomass being highest around seabirds: the posterior probability of a negative slope in the seabird-rich location was 97% (compared to 65% in the seabird-poor location) and was 94% for a more negative slope than for the seabird-poor location (figure 5b).

## 4. Discussion

Understanding how reef fishes respond to local environmental variability is important to predict how coral reefs may shift in a changing world. We show that seabird-derived nutrients are assimilated by cryptobenthic fishes close to seabird colonies, with observable differences in fish organismal traits. Specifically, the cryptobenthic fishes tested here showed elevated *δ*^15^N values, higher dependence on benthic carbon and larger sizes near seabird-enriched run-off, but this signal dissipated with increasing depth. This evidences that even in a fringing reef system constantly flushed by oceanic waves, point sources of seabird nutrients lead to direct, quantifiable changes in energy fluxes through fishes that are stationary enough to be exposed to these nutrients throughout their lives.

### (a) Seabird nutrient transfer to cryptobenthic fishes

The capacity of coral reef organisms to assimilate seabird-derived nutrients is well documented [1,10,13,14,18,63]. Among reef fishes, higher *δ*^15^N associated with seabird nutrient input has been demonstrated in the herbivorous damselfish *Plectroglyphidodon lacrymatus,* and several intertidal reef fishes also show seabird-driven ^15^N enrichment, including carnivorous and detritivorous blennies [1,24]. Our findings show that a similar effect is visible in several other short-lived fish species with mixed diets, as seabird nutrients were transferred across three families of cryptobenthic fishes. There was moderate to high evidence that this remained true when accounting for total length, suggesting that higher *δ*^15^N is not a result of increased fish size but indeed of seabird nutrient transfer to small fishes in this system. This is particularly notable given that the two sampling locations (La Cour beach and Anse Victorin) are only separated by approximately 1.5 km. The site-restricted nature of cryptobenthic fishes [44] may facilitate the stark differences in isotopic profiles we observed and suggests that the effects of localized nutrient inputs are not dampened in cryptobenthic reef fishes as opposed to other more mobile functional groups.

The clear differences in N isotope composition in the candidate sources we sampled provide important insights into how these nutrients may be transferred to cryptobenthic fishes. The filter-feeding sponge showed near-identical *δ*^15^N values in both locations, demonstrating a lack of seabird-nutrient enrichment in pelagic sources compared to the benthic source. In our system, benthic and pelagic pathways are therefore differentially affected by seabird presence, which appears to be reflected in some low-level consumers as well: seabird nutrient input is accompanied by an overall increase in *δ*^13^C in *Ev. guttata*, indicating an increase in the proportion of seabird-enriched, benthic food sources that are ingested near seabirds. Mixing models also suggest that cryptobenthic fishes switch to a higher proportion of seabird-enriched benthic food sources in shallow waters in close proximity to seabird colonies, with up to approximately 20% increase in the case of *En. abeli* and to a lesser extent for *Ev. guttata*. This suggests that cryptobenthic fishes across different feeding groups respond to point sources of allochthonous nutrients within a relatively narrow range from the nutrient source, by specifically displaying higher reliance on seabird-enriched resources.

### (b) Species-specific responses to nutrient enrichment

Contrary to our expectations, we did not find strong community-level responses to seabird nutrients in cryptobenthic fishes. Previous studies investigating cryptobenthic fish responses to environmental gradients showed strong local effects of disturbances or environmental conditions on cryptobenthic fish community composition [37]. Why species composition remains comparable across the seabird-rich and seabird-poor locations may be related to the small spatial scale separating the sampling locations (approx. 1.5 km). Such little distance between our sampling locations implies a high likelihood of larval exchange between the seabird-rich and the seabird-poor location, especially given cryptobenthic fishes’ short dispersal ranges and near-complete reef retention of larvae [64]. Both locations also present comparable exposure and habitats, which results in very little differences in environmental or biotic post-recruitment selection, ultimately leading to highly similar fish communities. An additional reason explaining the lack of changes in species composition resides in the time scale of such changes. Although seabird nutrient subsidies can return to rat-eradicated islands within 16 years [63], full recovery of seabird-associated benefits may necessitate several decades. Rat removal occurred 23 years prior to sampling in Fregate Island, which suggests that although seabird nutrients have at least partially returned, there is a possibility that marine communities have not shifted accordingly yet. A final factor, which may have contributed to a lack of community-level differences, is the nature of the disturbances we observe. In the case of the 1998 bleaching events [65], loss of habitat was the main driver of community differences: loss of corals created new niches, spurring quick recolonization by cryptobenthic fish larvae that thrive in degraded reef configurations. Because of high microhabitat specialization, habitat degradation is bound to cause severe changes in cryptobenthic communities [66]. On the other hand, seabird presence does not necessarily imply changes in habitats and microhabitats: algal cover, rubble cover and structural complexity remained comparable across areas of varying seabird density in the absence of other disturbances [16]. Given that the main change introduced by seabirds is nutrient supply, diet plasticity appears as the primary mechanism through which species may persist, therefore allowing cryptobenthic fish, as opportunistic foragers, to withstand these changes (but see [67]).

Our species-level analysis revealed contrasting responses to changes in nutrient supply. *Enneapterygius abeli* and *Ev. guttata* both featured comparable depth-related trends of N and C uptake, but the lower *δ*^13^C values of *Ev. guttata* suggest higher overall reliance on a pelagic pathway as opposed to *En. abeli*. While this may be explained, in part, by their depth distribution, even individuals sampled at identical depths showed a comparable pattern. In addition, although *Ev. guttata* and *Ev. bipunctata* both showed a positive effect of seabird nutrients on size, *En. abeli* appeared to be largely unaffected by seabird subsidies. This highlights that, despite broadly comparable life-history traits, the mechanisms of nutrient uptake, resource use and allocation may differ among cryptobenthic fish species. Congeneric species of cryptobenthic fishes can present strong ecological differentiation, driven by diet and physiology [67], and the species-specific responses to seabird nutrient enrichment we observe probably lie at the intersection of both. For example, *Eviotas* are highly opportunistic foragers with relative microhabitat flexibility [68] and may switch to seabird-enriched resources more effectively than other genera. On the other hand, the fact that *En. abeli* showed little increase in total length despite evidence of seabird nutrient uptake suggests that body size may be more constrained in triplefins, perhaps owing to conflicting selection pressures [69].

### (c) Productivity

Biomass gain in areas adjacent to seabird colonies has been documented for all feeding groups, with the largest effect size associated with herbivore biomass [1]. Here, we also demonstrate the positive effect of seabirds on visually conspicuous fish community biomass. Our observations of seabird nutrient uptake and increased sizes among some species of cryptobenthic fishes suggest that these benefits might indeed be transferred to their predators. All three of the feeding groups that can predate cryptobenthic fishes (omnivores, carnivores and piscivores) showed a biomass increase near seabirds, suggesting that cryptobenthic fishes could represent an important link through which seabird-derived nutrients are transferred onto higher trophic levels.

Contrary to other feeding groups, herbivorous biomass increased specifically at shallow depths. Herbivores may respond strongly to benthic seabird fertilization via direct ingestion of enriched substrate such as detritus, biofilm or the epilithic algal matrix [19,24]; by contrast, for other feeding groups, the impact of added nutrients may be diluted by intermediate trophic steps, the possible ingestion of both enriched and non-enriched lower levels and subsequent trophic dampening for primary and secondary consumers, hindering our ability to detect seabird effects in higher trophic levels specifically at shallower depths [70]. The divergences in how herbivorous and other feeding groups respond to seabirds along a depth gradient can also be explained by their respective home range. Our study sites were limited to Fregate Island’s northeastern area, and all transects occurred within an approximately 5 km^2^ area. In coral reef fishes, home ranges scale with body size but not necessarily trophic level [71]. The most represented piscivore in our surveys was *Cephalopholis argus*, which has an estimated home range of 230 m^2^ to approximately 2 km^2^ [72]. Movement across depths could confound depth-related nutrient uptake mechanisms, resulting in biomass increase at the seabird-rich site but no discernible depth-related trend. On the other hand, the herbivore with the highest biomass in our surveys was *Acanthurus leucosternon*, with an estimated home range of only 17 m^2^ [73], followed closely by *Ctenochaetus striatus*, with a home range extending between 7 and 240 m^2^ [74]. Such high site fidelity suggests that uptake and subsequent release of these nutrients by herbivores may be constrained within nearshore areas and shallower waters. Importantly, this implies that dissemination of seabird-derived nutrients via spatially constrained herbivory could be complemented by carnivorous fishes spreading these nutrients across larger and deeper portions of the reef.

In turn, the total absence of biomass increase for feeding groups that rely more strongly on pelagic resources like planktivores or corallivores also indicates that these benefits do not affect fish communities universally. Concordantly, pelagic subsidies, as indicated by sponge samples, presented little to no seabird-nutrient enrichment in our system. This is not necessarily the case elsewhere: in the Chagos archipelago, Graham *et al.* note higher seabird-derived enrichment of sponge compared to turf in a study conducted across three atolls, and at Palmyra Atoll, McCauley *et al.* show elevated *δ*^15^N in sponges and zooplankton associated with seabird-rich native forests [1,11]. Both are lagoon systems with potentially long residence times and where nutrient accumulation may be significant [75,76], as opposed to a rapidly flushed fringing reef. Our findings therefore highlight the role of reef type and hydrodynamics in determining the recipients of nutrient enrichment and the associated effect (or lack thereof) on the productivity of different feeding groups.

Taken together, our findings suggest that, in a fringing reef system that is flushed by oceanic waters, high transfer efficiency directly from enriched benthic autotrophs to consumers and high site fidelity are likely to strengthen the integration of seabird-derived nutrients into reef productivity. In turn, seabird-derived nutrients are propagated through carnivorous species, which may carry them farther and deeper into reefs. Identifying the extent to which cryptobenthic fishes causally contribute to these benefits will require targeted sampling and experimentation.

## 5. Conclusion

Our results indicate that cryptobenthic fishes benefit from seabird-derived nutrients, altering their reliance on benthic versus pelagic nutrient pathways and impacting cryptobenthic fish species’ sizes, weights and, by extension, potential contributions to reef functioning. Thus, in a fringing reef system that is continuously flushed by oceanic waves, cryptobenthic fishes appear to be responsive to point sources of nutrients within a narrow band from the shore, and, as such, they may represent a path of trophic transfer for seabird-derived nutrients to higher trophic levels. Our findings also suggest that seabird nutrient subsidies may dissipate quickly with increasing depth. Benefits to herbivorous reef fishes, for which high trophic transfer efficiency, small home ranges and direct use of nutrient-enriched autotrophs result in strong responses even within a relatively narrow band of nutrient enrichment, are spatially limited and could therefore be complemented by cryptobenthic fishes and their predators. Our results further emphasize the importance of seabird conservation in small, isolated islands for which these nutrient sources are vital for maintaining and enhancing productivity. In the wider context of anthropogenic alteration of nutrient supplies, it remains crucial to understand to what extent variations in nutrient input affect reef fishes and how these effects can be mitigated to preserve ecosystem productivity.

## Data Availability

All data and code are available on Zenodo, accessible at [77]. Supplementary material is available online [78].
